# Interictal brain activity differs in migraine with and without aura: resting state fMRI study

**DOI:** 10.1186/s10194-016-0716-8

**Published:** 2017-01-25

**Authors:** Péter Faragó, Bernadett Tuka, Eszter Tóth, Nikoletta Szabó, András Király, Gergő Csete, Délia Szok, János Tajti, Árpád Párdutz, László Vécsei, Zsigmond Tamás Kincses

**Affiliations:** 1 Department of Neurology, Neuroimaging Research Group, Albert Szent-Györgyi, Clinical Center, University of Szeged, Semmelweis u. 6, H-6725 Szeged, Hungary; 2 MTA-SZTE Neuroscience Research Group, Szeged, Hungary; 3 International Clinical Research Center, St. Anne’s University Hospital Brno, Brno, Czech Republic

**Keywords:** ALFF, Frequency analysis, Migraine, Migraine with aura, MRI

## Abstract

**Background:**

Migraine is one of the most severe primary headache disorders. The nature of the headache and the associated symptoms during the attack suggest underlying functional alterations in the brain. In this study, we examined amplitude, the resting state fMRI fluctuation in migraineurs with and without aura (MWA, MWoA respectively) and healthy controls.

**Methods:**

Resting state functional MRI images and T1 high-resolution images were acquired from all participants. For data analysis we compared the groups (MWA-Control, MWA-MWoA, MWoA-Control). The resting state networks were identified by MELODIC. The mean time courses of the networks were identified for each participant for all networks. The time-courses were decomposed into five frequency bands by discrete wavelet decomposition. The amplitude of the frequency-specific activity was compared between groups. Furthermore, the preprocessed resting state images were decomposed by wavelet analysis into five specific frequency bands voxel-wise. The voxel-wise amplitudes were compared between groups by non-parametric permutation test.

**Results:**

In the MWA-Control comparison the discrete wavelet decomposition found alterations in the lateral visual network. Higher activity was measured in the MWA group in the highest frequency band (0.16–0.08 Hz).

In case of the MWA-MWoA comparison all networks showed higher activity in the 0.08–0.04 Hz frequency range in MWA, and the lateral visual network in in higher frequencies.

In MWoA-Control comparison only the default mode network revealed decreased activity in MWoA group in the 0.08–0.04 Hz band.

The voxel-wise frequency specific analysis of the amplitudes found higher amplitudes in MWA as compared to MWoA in the in fronto-parietal regions, anterior cingulate cortex and cerebellum.

**Discussion:**

The amplitude of the resting state fMRI activity fluctuation is higher in MWA than in MWoA. These results are in concordance with former studies, which found cortical hyperexcitability in MWA.

## Background

Migraine is a common disabling disease, affecting about 10% of the population [1]. While not life threatening, it has a significant effect on the quality of life. Two major forms of the disease present with or without transient, focal neurological symptoms, called aura [2]. While the pathomechanism of migraine is not entirely understood, the two subgroups of the disease thought to have different background [3, 4]. It was suggested that cortical spreading depression, a slow depolarization wave traveling anteriorly in the brain is responsible for the aura symptoms and occur exclusively in migraine with aura (MWA) [5, 6]. Cortical hyperexcitability was mentioned as the potential trigger of migraine [7–12] and this hyperexcitability is also present in MWA more robustly [13].

Information about the resting brain activity can be noninvasively gathered by BOLD fMRI. Traditionally fMRI studies compare signal in various phases of a task, but acquiring BOLD signal in rest, allows the studying of resting brain activity fluctuations. Interestingly, remote areas show synchronous activity, which renders resting state activity into functional networks [14, 15].

Several studies investigated the activity of the resting state functional networks in migraine and found various alterations of networks that are implicated in pain processing [16–19]. Furthermore, a few studies investigated patients with MWA and migraine without aura (MWoA) and reported various aspects of altered connectivity in the subgroups of the disease [20–25]. Remarkably some of the studies found increased functional connectivity, in comparison to decreased functional connectivity in migraine.

Most of the resting state fMRI studies investigated the connectivity between various regions and therefore build on the coherent activity in spatially distributed networks. The variation in the frequency and the amplitude of the resting state BOLD signal is usually neglected. The BOLD resting state fluctuation is a low frequency fluctuation [14, 15]. To filter out the non-neural noise from the raw BOLD time courses most of the studies apply filters [16, 26, 27]. However, neural signal could be detected also in the higher frequencies [28]. Furthermore, a few recent studies started to analyse the amplitude of the low frequency fluctuation of the resting state signals [29, 30] offering a unique insight into the resting brain activity in various diseases but not in migraine.

Based on the above described premises, the increased cortical excitability in migraine might be related to the frequency specific alteration of amplitude of the resting BOLD activity. MWA and MWoA being different in respect to cortical excitability might also appear in the differential frequency spectrum of the resting brain activity in the two subgroups of the disease.

In the current study we investigated the resting state BOLD fluctuations in migraine with special focus on the amplitude and the frequency of the activity.

## Methods

### Participants

Fifty-three patients with migraine were recruited into this study from the Headache Outpatients Clinic, at the Department of Neurology, University of Szeged. All of the patients were diagnosed with episodic migraine and were scanned during the interictal phase, having had the the scanning at least one week to the last attack. The diagnosis set up by the International Classification of Headache Disorders [2]. Eighteen patients suffered from MWA (17 visual aura, 1 sensory aura), the other 35 patients never experienced aura. Patients had no other neurological or psychiatric disorders.

Thirty-two healthy volunteers were recruited. None of the controls had any records of any neurological or psychiatric disorders. For demographic data of patients and controls, see Table 1.

**Table 1:** Demographic data ATK/life: estimated headache attacks over lifetime. ATK/years: annual headache attack frequency. There were no significant difference between the groups in terms of age (p<0.82) or gender (p<0.8). There were no significant difference in the disease duration (p<0.65) or allodynia score (p<0.2) between migraine groups. There were significant differences between the two patient group in VAS (p<0.0.3) and yearly attacks (p<0.03)

	Migraine with aura	Migraine without aura	Healthy
*n*	***18***	***33***	***32***
age (years; mean and SD)	***32.1(8)***	***35.6(8.9)***	***35.2(11)***
gender (male)	***3***	***3***	***2***
Allodynia	***1.6(1.7)***	***3.2(3)***	***NA***
Disease duration (years; mean and SD)	***14.2(8.6)***	***13.7(9.1)***	***NA***
ATK/life (days; mean)	***461(615)***	***656(626)***	***NA***
VAS	***7.6(1.3)***	***8.7(1.2)***	***NA***
ATK/years (days; mead and SD)	***29(26)***	***55(45.6)***	***NA***

The study was approved by the ethics committee of the University of Szeged and all study participants gave written informed consent in accordance with the Declaration of Helsinki (authority number: 56/2011).

### Image acquisition

The MR imaging was performed on a 1.5 T GE Signa Excite HDxt MRI Scanner (Milwaukee, WI, USA). The head motion was restricted with foam padding around the head and the noise of the scanner was attenuated with earplugs. For every participants high-resolution T1 weighted images (3D IR-FSPGR: TR/TE/TI: 10.3/4.2/450 ms, flip angle: 15°, ASSET: 2, FOV: 25*25 cm, matrix: 256*256, slice thickness: 1 mm,) and a resting state fMRI protocol with echo-planar imaging technique (TE: 40 ms, TR: 3000 ms, matrix: 64*64 cm, FOV: 30*30 cm, slice thickness: 6 mm, flip angle: 90°, NEX: 1, ASSET: 2,0 Ph, Phases per Loc: 128, volumes: 200) were acquired. Subjects were asked to be awake during the acquisition eyes closed.

### Data processing

All image processing were performed by FMRIB’s Software Library (http://www.fmrib.ox.uk/fsl, Oxford, UK) toolkits.

### Preprocessing

The pre-processing was carried out with FEAT (FMRI Expert Analysis Tool). The first two images were removed from all resting state datasets. The non-brain parts were removed using Brain Extraction (BET) [31]. Motion correction (MCFLIRT) [32] were applied in all images and spatially smoothed with Gaussian kernel of 6 mm FWHM. The high-pass filter cut-off was applied to all functional images (with sigma 100 s).

All pre-processed resting state images were registered to standard space (MNI152 T1 image; 2 mm slice thickness) and to their high-resolution T1 images with linear (FLIRT) and then with non-linear registration (FNIRT) [32]. All images were resampled to 4 mm isovoxels.

### Independent component analysis

Resting state networks were identified by group independent component analysis as implemented in the MELODIC toolbox, part of the FSL software library (FMRIB Software Library, htttp://www.fmrib.ox.uk/fsl) [15]. The individual preprocessed and standard space registered functional images were concatenated after voxel-wise de-meaning and variance normalization. The 4D data were decomposed into a set of matrices that characterize the underlying processes in the spatial and temporal domains in such a way that spatial matrices are maximally non-Gaussian. The number of components was estimated by applying the Laplace approximation to the Bayesian evidence of a probabilistic principal component model [33]. Spatial maps were processed by using an alternative hypothesis test based on the fitting a Gaussian/Gamma mixture model to the distribution of voxel intensities within spatial maps and a posterior probability threshold of *p* > 0.5. The resulting subject-wise time courses are to be understood as the temporal characteristics of the network activity across the entire spatial map for each individual subject.

### Amplitude of resting state activity

The frequency specific modulation of the amplitude of resting activity was analysed by discrete wavelet decomposition (Wavelet Toolbox of the Matlab software package; MathWorks Inc). The networks’ time courses were divided into five consecutive frequency bands (band1: 0.16–0.08 Hz, band2: 0.08–0.04 Hz, band3: 0.04–0.02 Hz; band4: 0.02–0.01 Hz; band5: 0.01–0 Hz) using discrete wavelet decomposition. Discrete wavelet decomposition is an implementation of the wavelet transform using a set of predefined wavelet scales and translations and decomposes the signal into mutually orthogonal set of wavelets. Wavelets are brief waves, they are finitely extended and their oscillations decay to zero rapidly, satisfying the admissibility condition:

∫Ψ(*t*)*dt* = 0.

By dilating and translating a “mother” wavelet (Ψ) and a “father” wavelet (Φ) (∫Φ(*t*)*dt* = 1) a wavelet family can be obtained:

$$ {\Psi}_{jk}(t)=\frac{1}{\sqrt{2^j}}\Psi \left(\frac{t-{2}^jk}{2^j}\right); $$

$$ {\Phi}_{jk}(t)=\frac{1}{\sqrt{2^j}}\Phi \left(\frac{t-{2}^jk}{2^j}\right); $$

where j is index of the scale *S*_*j*_ *= 2* ^*j*^ and k indexes the *K = n/2* ^*j*^ location in time. The Daubechies wavelet was used as mother wavelet. The analysis by using a halfband filtering, decomposes the data over a hierarchy of scales (*S*_*j*_). At each scale the data is split into two orthogonal components: details (*d*_*jk*_) containing the high frequency information and approximations (*a*_*jk*_) containing the low frequency information [34]. By five levels of decomposition the following frequency bands were derived 0–0.16 Hz: 0.16–0.08 Hz, 0.08–0.04 Hz, 0.04–0.02 Hz, 0.02–0.01 Hz and 0.01–0 Hz.

To measure the amplitude of the resting activity in the various frequency bands an envelope was fitted to the absolute values of each frequency bands: The minimums of the following function was identified:

$$ f(t)=\frac{d\left(\operatorname{sgn}\left(\frac{dy}{dt}\right)\right)}{dt} $$

and a linear interpolation of these points were used to create an envelope. The envelopes were averaged over time to describe the mean activity in the given frequency band.

The analysis outlined above was applied to (i) the mean activity of the ICA identified resting state networks and (ii) to the voxel-wise preprocessed fMRI data.

i. To compare the amplitude of the mean network timecourses across groups in the individual frequency bands General Linear Model (GLM) based test was used. Gender and age were included in our analysis as covariate.
ii. For voxel-wise comparison of the amplitude of the resting activity across groups a nonparametric permutation test was performed (5000 permutations) for each frequency bands. The design encoded for group membership, age and gender were used as nuisance variables. For statistical analysis threshold-free cluster enhancement were used (TFCE) and corrected for multiple comparisons (across space) within the permutation framework. Age and gender also included in this analysis as nuisance variable.

Additionally, we performed the voxel wise comparison of the resting state networks’ for the non-filtered data also.

## Results

### Demographical data

There were no significant difference between the groups’ age or gender distribution. There were no significant differences between the two migraineus group in disease duration.

### Amplitude of the activity of the resting state functional networks

A MELODIC analysis found 33 components in the healthy controls. The artefact components were removed from our analysis based on previous studies [14, 15] and five networks were included for analysis: default mode network, right attention network, left attention network, medial visual network, lateral visual network.

### MWA vs. healthy controls

There were no significant differences between the amplitude of the resting activity in MWA vs. healthy controls. While not significant, in the highest frequencies (0.16–0.08 Hz) the amplitude of the resting activity was slightly higher in the left attention network (*p* = 0.07) and in the right attention network (*p* = 0.059) in MWA as compared to healthy controls.

### MWA vs. MWoA

**Fig. 1:**
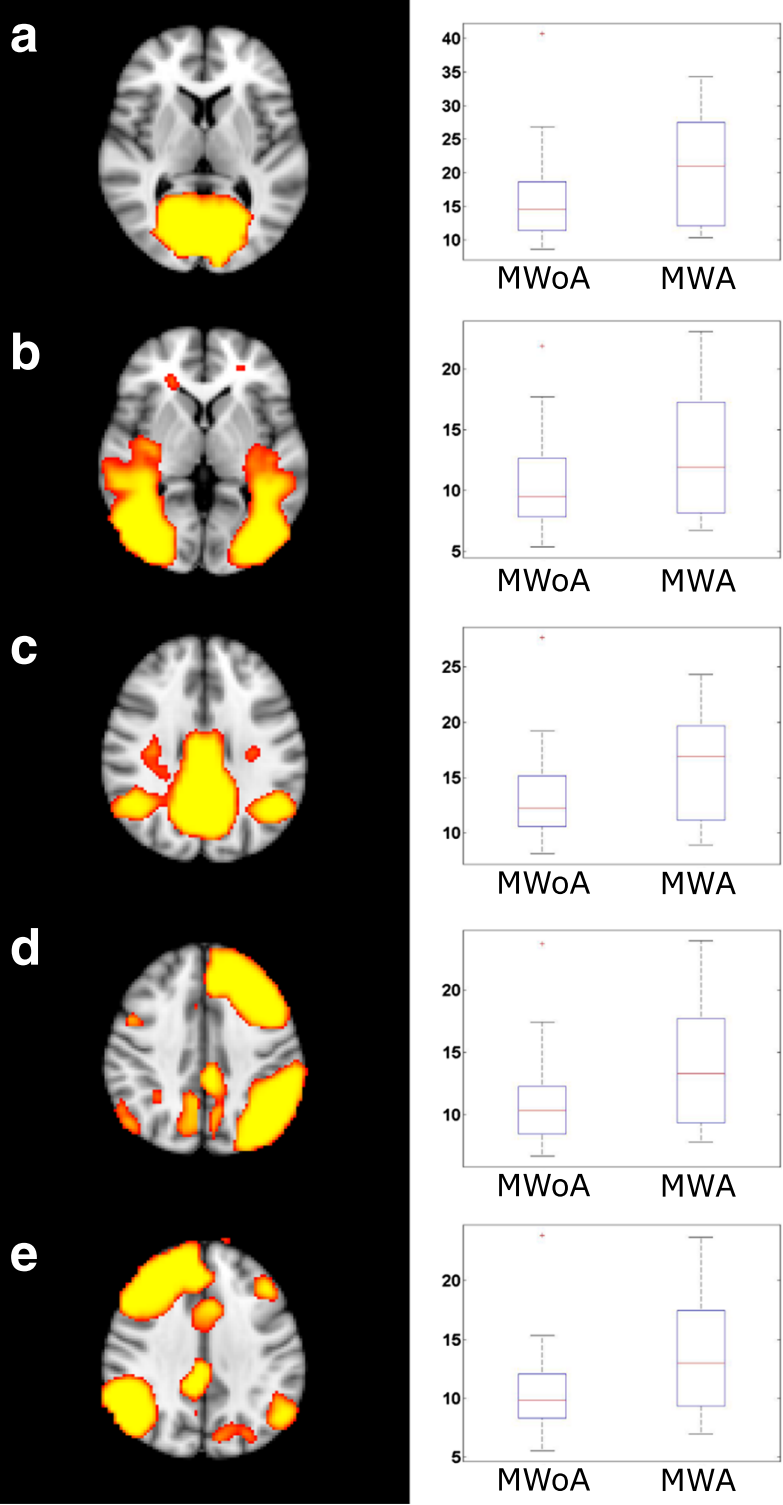
All of the investigated network ((**a**) medial visual, (**b**) lateral visual, (**c**) default mode, (**d**) right attention, (**e**) right attention) showed higher amplitude in the 0.08–0.04 Hz frequency range in MWA compared to MWoA. The images on the left depicting the resting state networks are thresholded at *p* < 0.5 and overlaid on the standard MNI_152 brain. The boxplots depicting the amplitude of the activity of the networks in the 0.08-0.04 Hz frequency range. The central mark is the median, the edges of the box are the 25th and 75th percentiles, the whiskers extend to the most extreme datapoints

The amplitude of the activity was higher (*p* < 0.05) in all examined networks in the 0.08–0.04 Hz frequency range and in the lateral visual network also in the 0.16–0.08 Hz frequency band (Fig. 1 *)* in the MWA group*.* There were no other significant results in any other frequency bands or in case of the non-filtered data.

### MWoA vs. Healthy controls

**Fig. 2:**
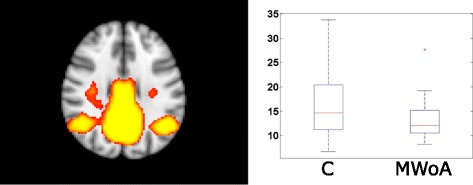
The default mode network showed higher amplitude in the 0.04–0.02 Hz in healthy group compared to MWoA. The images on the left depicting the resting state networks are thresholded at *p* < 0.5 and overlaid on the standard MNI_152 brain. The boxplots depicting the amplitude of the activity of the networks. The central mark is the median, the edges of the box are the 25th and 75th percentiles, the whiskers extend to the most extreme datapoints

The amplitude of resting activity in the 0.08–0.04 Hz range was lower in patients without aura in the default mode network (*p* < 0.05) (Fig. 2 *).* There were no other significant results in any other frequency bands or in case of the non-filtered data.

### Voxel-wise comparison of the amplitude of resting activity

**Fig. 3:**
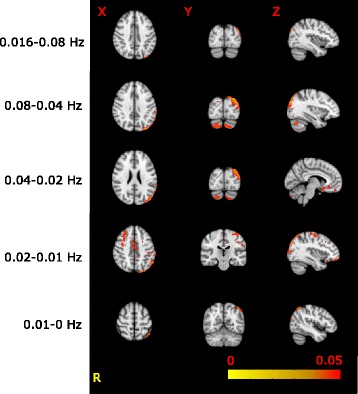
The frequency specific voxel wise comparison of the amplitude of the resting activity showed higher amplitudes in MWA as compared to MWoA in all freqeuncy ranges. The voxel wise changes in each frequency range. The images are thresholded at *p* < 0.05 corrected for multiple comparisons and overlaid on the standard MNI_152 brain. We marked the sidedness with 'R' as the right side. The X-Y-Z letters indicates the axes of the pictures above them

The voxel-wise comparison of the amplitude of resting activity showed higher amplitudes (*p* < 0.05, corrected for multiple comparisons) in the left parietal lobe in all frequency ranges in MWA compared to MWoA. In addition, in the 0.08–0.04 Hz frequency band the amplitudes were higher in the bilateral cerebellum, in the left occipital pole and occipito-temporal junction, and a smaller cluster was found in the right inferior parietal lobule. In the 0.04–0.02 Hz frequency band the amplitudes were higher in MWA compared to MWoA in the left inferior parietal lobule, bilateral cerebellum and in the anterior cingulate gyrus. In the 0.02–0.01 Hz range next to the inferior parietal lobule, occipital pole and cingulate gyral differences amplitudes were found higher in the bilateral frontal lobe around the superior frontal sulcus and precentral gyrus (Table 2 and Fig. 3).

**Table 2:** Increased amplitude of resting state activity fluctuations in MWA as compared to MWoA

	Region	side	x	y	z	*p*<
0–0.16 Hz	Inferior parietal lobule	L	-38	-82	40	0.03
0.16–0.08 Hz	Inferior parietal lobule	L	-34	-84	42	0.02
	Cerebellum	L	-6	-84	-40	0.04
	Occipito-temporal junction	L	-58	-70	0	0.03
	Occipital pole	L	-16	-96	14	0.05
	Inferior parietal lobule	R	14	-84	34	0.05
	Cerebellum	R	36	-34	-52	0.05
0.08–0.04 Hz	Cingulate gyrus	L	-6	14	-18	0.04
	Inferior parietal lobule	L	-34	-84	42	0.03
	Cerebellum	R	24	-84	-40	0.04
	Cerebellum	L	-14	-80	-46	0.05
0.04–0.02 Hz	Inferior parietal lobule	L	-34	-84	42	0.02
	Superior frontal sulcus	R	26	34	44	0.04
	Precentral gyrus	L	-44	-12	62	0.04
	Frontal pole	L	-34	52	-16	0.03
	Cingulate gyrus	L	0	-2	42	0.05
	Occipital lobe	L	-22	-92	12	0.04
0.02–0.01 Hz	Inferior parietal lobule	L	-44	-60	58	0.03

There were no differences between the MWoA and healthy group or between the MWA and control group.

## Discussion

This study identifies novel frequency specific alterations of resting fluctuation of fMRI measured brain activity. Our analysis showed that amplitude of resting state BOLD fluctuation is higher in MWA in the cingulate cortex, superior parietal lobule, cerebellum and bilateral frontal regions compared to MWoA. Furthermore, amplitude of the activity of the resting state networks in the 0.08–0.04 Hz frequency range was higher in MWA than in MWoA in all examined resting state networks. In MWoA the amplitude of the activity fluctuation of the default mode network was lower than in healthy controls.

Several lines of evidence supports the hyperexcitability of the cortex in migraine [7–12]. The amplitude of visual evoked potentials (VEP) were showed higher in migraineurs [11, 34–36]. However, recent reports showed that the VEP measured hyperexcitability predominantly true for migraine with aura [37, 38].

The threshold of transcranial magnetic stimulation evoked phosphenes are also lower in migraineurs and the prevalence of phosphenes are higher [9]. Interestingly, recent metaanalysis pointed out that, similar to the VEP results, this kind of TMS measured hyperexcitability is not evident for MWoA and only true for patients experiencing aura [39].

Similarly, neuroimaging PET and fMRI studies found higher activation for visual stimuli in migraineurs [40–42], but recent reports showed that patients with aura show higher BOLD response to photic stimuli in contrasts to those migraineurs not experiencing aura symptoms [43, 44].

A possible explanation of such interictal hyperexcitability in migraine might be the higher interictal glutamate/glutamine ratio [45] or the lower gamma-amino butyric acid level [46] in the occipital cortex as shown by MR spectroscopy studies. A recent investigation also showed larger reduction of N-acetyl-aspartate levels in a functional MR spectroscopy study in MWA but not in MWoA [47] indicating a less effective mitochondrial functioning in MWA.

These above mentioned studies, indicating cortical hyperexcitability in MWA are in line with our current results. The amplitude of the resting BOLD fluctuation was higher in MWA patients in regions highly related to pain processing and in certain frequencies, the amplitude of the activity of the resting state networks was also higher. While there is no direct evidence that various electrophysiological measures of cortical excitability are represented as a variation in the amplitude or the frequency of the resting BOLD fluctuations, one might speculate that they are strongly related and both depict important features of cortical function.

Several researches investigated the brain resting fMRI activity in migraine with various approaches [17, 19–21, 23, 43, 48, 49]. These approaches are not giving direct information on the cortical hyperexcitability, but a measure of functional interaction. A few studies investigated the amplitude of the resting state BOLD fluctuation in chronic pain conditions [50, 51] and found higher amplitude of resting activity in chronic back pain, irritable bowel syndrome, knee osteoarthritis and complex regional pain syndrome. Only single study investigated the amplitude of the low frequency fluctuations in 24 migraineurs, without grouping the patients based on the aura symptoms [52]. Concentrating on only the low frequency component of the BOLD signal (0.01–0.08 Hz) they found lower amplitude in migraineus in the cerebellum, bilateral frontal and occipital regions and increased amplitudes in the brainstem and insula. Also our results sustain the well known importance to investigate patients with and without aura symptoms in separate groups. Only the patients experiencing aura symptoms were the ones who had higher amplitude BOLD fluctuation in our study. This was true for the amplitude of the resting activity and also for the amplitude of the activity of the resting state networks.

The importance of the various frequencies of BOLD fluctuations is not yet known but recently a few studies started to explore this feature [53], especially in pain conditions [30, 54]. It was proposed that functional connectivity of various brain regions are represented in different dominant frequency bands [55]. Another option might be that migraine was proposed to be a neurovascular disease, and the altered neurovascular coupling may affect the frequency of the resting BOLD fluctuations [56] by acting as a filter. Furthermore, the group difference in the resting state network activity might well be the result of improved signal to noise ratio by filtering out the low and high frequency artifact. Since most of the slow frequency fluctuation in our analysis with a relatively long TR were shown to be neural origin [57], this hypothesis seems rather unlikely.

Another important aspect of our results is the spatial localisation of the amplitude differences. In the various frequency ranges extensive fronto-parietal and cerebellar areas showed higher activity in MWA patients. Most of these regions were associated with various aspects of the pain processing. Primary and secondary somatosensory cortices, insula, cingulate cortex and prefrontal cortex and subcortical regions, such as the periaqueductal gray matter, hypothalamus, amygdala, hippocampus and cerebellum are commonly activating in response to painful stimuli and referred to as the pain matrix [58]. These key regions were reported to subserve multiple functions in pain processing, including sensory discrimination, motivation-affect, motor, attention and also arousal and response selection functions [59].

Cingulate cortex in particular was shown to be one of the key region in experiencing pain [60–62] and it is also part of the descending pain modulatory system [63]. Anterior cingulate cortex is connected to the periaqueductal grey matter, a key region in migraine pathomechanism [64]. In a recent work we also showed that chronic inflammatory pain related sensitization is mediated by the altered activation and connectivity of the cingulate cortex in rats [65].

It was also shown that cerebellum actively contributes to various aspects of pain processing [66]. Subclinical vestibulo-cerebellar dysfunctions were found in migraine with and without aura [67, 68]. The macro and microstructure of is cerebellum is altered in migraine [69, 70]. The interictal perfusion of the cerebellum was not found affected by the disease, independently of aura symptoms [71]. In migraine patients Wang and colleagues found altered amplitude of low frequency fluctuation [52], higher and lower amplitudes were found in various parts of the cerebellum. Importantly, in their study no information was given if the patients experienced aura.

The fronto-parietal regions showing increased amplitude of resting activity fluctuation in our study are presumably the same, which are considered to be associated with executive functions. The subclinical executive dysfunction is a known feature of the disease [49, 72, 73]. A recent investigation also described the altered expression of the fronto-parietal resting state network in MWoA patients [49] in highly similar regions what we have found in our analysis.

## Conclusion

In summary, the the regions we found to have increased amplitude of activity fluctuation in MWA patients are those known to be affected by the disease and responsible for the functional alterations also.
